# An Internal Real-Time Microscopic Diagnosis of a Proton Battery Stack during Charging and Discharging

**DOI:** 10.3390/ma16093507

**Published:** 2023-05-02

**Authors:** Chi-Yuan Lee, Chia-Hung Chen, Chin-Yuan Yang, Wan-Ting Chen

**Affiliations:** 1 Department of Mechanical Engineering, Yuan Ze Fuel Cell Center, Yuan Ze University, Taoyuan 32003, Taiwan; 2 HOMYTECH Global Co., Ltd., Taoyuan 33464, Taiwan

**Keywords:** proton battery stack, micro-electro-mechanical systems, flexible seven-in-one microsensor, charge/discharge experiment

## Abstract

The proton battery has facilitated a new research direction for technologies related to fuel cells and energy storage. Our R&D team has developed a prototype of a proton battery stack, but there are still problems to be solved, such as leakage and unstable power generation. Moreover, it is unlikely that the multiple important physical parameters inside the proton battery stack can be measured accurately and simultaneously. At present, external or single measurements represent the bottleneck, yet the multiple important physical parameters (oxygen, hydrogen, voltage, current, temperature, flow, and humidity) are interrelated and have a significant impact on the performance, life, and safety of the proton battery stack. This research uses micro-electro-mechanical systems (MEMS) technology to develop a micro oxygen sensor and integrates the six-in-one microsensor that our R&D team previously developed in order to improve sensor output and facilitate overall operation by redesigning the incremental mask and having this co-operate with a flexible board for sensor back-end integration, completing the development of a flexible seven-in-one (oxygen, hydrogen, voltage, current, temperature, flow, and humidity) microsensor.

## 1. Introduction

The acceleration of global warming has forced various countries to accelerate the development of energy sources that can substitute fossil fuels, and it is hoped that renewable energy can be used in people’s livelihoods and in industrial conditions as soon as possible. At present, the most urgent goal of the world is to achieve zero carbon emissions by 2050. Hydrogen energy is characterized by high-efficiency generation, low noise, and water as the only byproduct, meaning that various countries have accelerated the development of hydrogen-related industries in recent years. The hydrogen energy-related market, e.g., fuel cells, is expected to reach a market scale of USD 52 billion by 2027 and has great advantages and development potential in the future [1]. With an increasing demand for renewable energy, large-scale energy storage equipment is receiving unprecedented attention because it can integrate intermittent renewable energy into the grid. One of the key challenges lies in the rapid change and daily fluctuations of renewable energy, so energy storage equipment is required for a quick response to mitigate the rise or fall in energy [2].

In recent years, the research on proton batteries has concentrated on the treatment of hydrogen storage materials, and activated carbon is usually used as the research subject. The hydrogen absorption capacity of activated carbon is proportional to its specific surface area (Brunauer, Emmett, and Teller). Therefore, related studies often focus on the treatment and activation of activated carbon [3].

As an attractive charge carrier, protons have received extensive attention. Because it has the smallest ion size and the lightest weight, it is almost better than all other cations [4]. Proton-conducting electrolytes are mainly used in batteries, proton exchange membrane fuel cells, supercapacitors, and photoelectrochemical and electrochromic devices [5,6]. This type of electrolyte is very useful for creating organized energy storage systems, and these electrolyte-based batteries have a longer battery life, higher performance, easier maintenance, and energy characteristics that can bear different stages of operation [7,8]. Multiple cathode and anode materials have been proven to be applicable to proton storage in acid electrolytes [9,10,11]. The proton battery has become a promising innovative energy storage technology for rich hydrogen, low pollution, and high safety. It is noteworthy that the rapid long-distance transmission of protons can be implemented by the rupture and recombination of covalent bonds and hydrogen bonds in the hydrogen network [12]. In addition, the light load mass of protons allows for a lower transmission potential barrier, meaning that the proton battery can achieve high energy and power density simultaneously, even at low temperatures [13,14]. The proton battery is generally regarded as a substitute for lithium-ion batteries because the H^+^ ion has a smaller radius than the Li^+^ ion [15]. The electrolyte directly reacting with the electrode in the battery is related to battery safety, time stability, energy density, and power density [16]. The proton battery is a novel secondary battery that uses protons instead of metallic ions as charge carriers. It consists of Faraday electrodes and acid electrolytes. The charge radius of H^+^ is obviously smaller than the charge radius of other ions, so the ion migration is faster. In addition, replacing the high-cost Li^+^ with cheaper and richer H^+^ provides a better option for environmentally friendly and inherently safe energy storage [17,18]. Li et al. [19] indicated that when a proton exchange membrane water electrolyzer produces hydrogen, it causes higher activity in the water molecules in the air, meaning system performance degrades faster under a high applied potential. Therefore, this study used micro-electromechanical systems (MEMS) technology to develop a flexible seven-in-one microsensor that could be embedded in a proton battery stack for charge/discharge experiments to measure the performance of the stack and extract real internal data.

Prior to this, our laboratory preliminarily developed proton battery single cells and proton battery stacks. This research aimed to improve battery performance based on proton battery stacks and test the hydrogen absorption capacity of activated microporous carbon, adjusting the temperature to test the optimal temperature conditions for the operation of the proton battery stack. In this study, a flexible seven-in-one micro-sensor was developed to embed within the proton battery stack to capture changes in seven different physical quantities (oxygen, hydrogen, temperature, humidity, flow, voltage, and current).

## 2. Design and Process of Flexible Seven-in-One Microsensor

**Figure 1:**
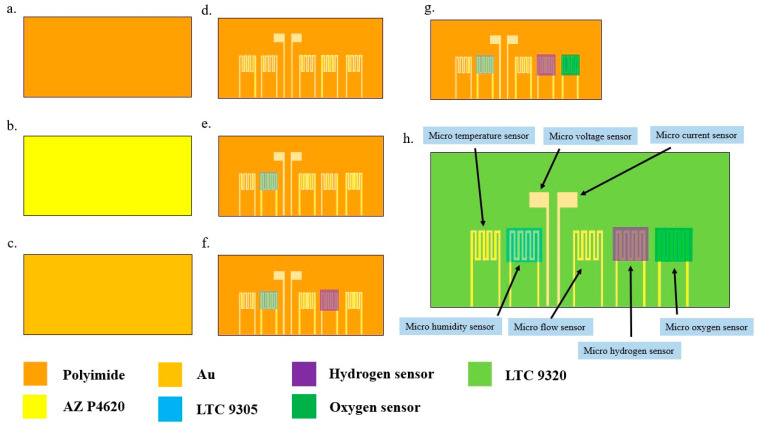
Fabrication process for the flexible seven-in-one microsensor. (**a**) Clean Polyimide (PI) film; (**b**) AZP 4620 was spin-coated on PI film to define the pattern; (**c**) Evaporate Ti as the adhesion layer of the Au and PI films to increase the adhesion of the Au and PI films; (**d**) Spin-coat LTC 9320 on the flexible seven-in-one microsensor to complete the protective layer; (**e**) Spin-coat LTC 9305 on the humidity sensor to complete the dielectric layer; (**f**) Sequential evaporation of Cr and Au followed by spin-coating of AZP 4620 on micro pressure sensors; (**g**) AZP 4620 was spin-coated on the micro hydrogen sensor, followed by evaporation of SnO_2_ to a thickness of 150 Å and Pt to a thickness of 25 Å; (**h**) AZP 4620 was spin-coated on the micro oxygen sensor, and then Zn was va-por-deposited to a thickness of 25 Å using an electron beam evaporator as a catalyst for the micro oxygen sensor.

In order to implement batch production to reduce cost, this study reduced the size of the sensing head of the proposed seven-in-one microsensor (voltage, current, temperature, flow, humidity, hydrogen, and oxygen) in comparison to the prior microsensor. For the developed microsensor and sensing area, as shown in Table 1, a serpentine design was used for the microsensors, except for the voltage and current microsensors, improving the resistance, and the serpentine conductor width is 10 μm. The fabrication process is shown in Figure 1. The substrate of the flexible seven-in-one microsensor was made of polyimide (PI) with high strength, high-temperature resistance, stability, and good chemical characteristics.

(a) First, the PI film was cleaned with acetone and organic methanol solutions, respectively, followed by deionized water to rinse the substrate surface with chemical solvents;
(b) AZP 4620 was spin-coated on PI film to define the pattern;
(c) Use an electron beam evaporation machine to evaporate Ti as the adhesion layer of the Au and PI films to increase the adhesion of the Au and PI films. Evaporate Ti with a thickness of 150 Å at a rate of 0.1 Å/s as the adhesion layer, and then evaporate 1500 Å-thick Au.
(d) Spin-coat LTC 9320 on the flexible seven-in-one microsensor to complete the protective layer.
(e) Spin-coat LTC 9305 on the humidity sensor to complete the dielectric layer.
(f) Evaporate 150 Å-thick Cr and 1500 Å-thick Au sequentially at a rate of 0.1 Å/s, and then spin-coat AZP 4620 on the micro pressure sensor to complete the upper electrode of the micro pressure sensor.
(g) Spin-coat AZP 4620 on the micro hydrogen sensor, and then use an electron beam evaporation machine to vapor-deposit SnO_2_ to a thickness of 150 Å and Pt to a thickness of 25 Å at a rate of 0.05 Å/s as the catalyst for the micro hydrogen sensor. For the material, the fabrication of the micro hydrogen sensor is complete.
(h) Spin-coat AZP 4620 on the micro oxygen sensor, and then use an electron beam evaporation machine to vapor-deposit Zn to a thickness of 25 Å as a catalyst for the micro oxygen sensor on the substrate to complete the fabrication of the micro oxygen sensor.

## 3. Internal Real-Time Microscopic Diagnosis of Proton Battery Stack during Charging

Before the actual charge/discharge of the proton battery stack, the activated carbon should be stored in the proton battery stack for hydrogen absorption. In this study, the activated carbon was stored in the 3 mm modified carbon felt located at the hydrogen ends on both sides, with 0.4 g on each side; the total weight of the activated carbon was 0.8 g. As most of the commercially available activated carbon is mixed with large particles, the activated carbon in this study was filtered through a 200 mesh screen before being placed in the proton battery stack. Afterward, the proton battery stack was assembled, and 20 mL of 2 M sulfuric acid was dripped into the sulfuric acid storage area so that the gas passages in the sulfuric acid storage area were filled with sulfuric acid for convenient gas ionization.

### 3.1. Proton Battery Stack Charge Experiment

**Figure 2:**
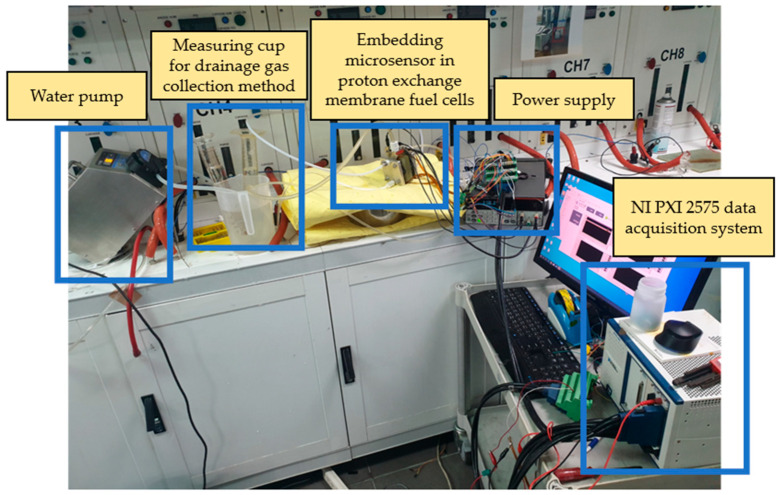
Instrument layout of the charge experiment for the proton battery stack.

**Figure 3:**
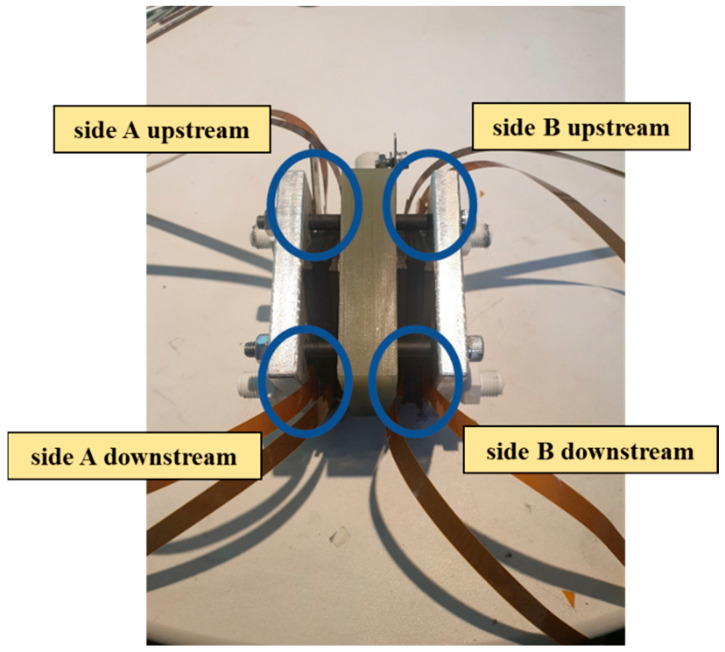
Entity diagram of the proton battery stack with a microsensor.

The proton battery stack was charged at 30 °C, 40 °C, and 50 °C to study the charging and hydrogen absorption capacities. During charging, the upstream water hole on the B side of the oxygen end was connected to the downstream water hole on the A side of the oxygen end by a water pipe. The upstream and sulfuric acid storage area on the A side of the oxygen end was connected using pipes, which were individually inserted into an inverted measuring cup filled with DI water (deionized water) for drainage and gas collection. Finally, the downstream water hole on the B side of the oxygen end was connected to a flow pump. The power supply provided 0.1 A current for the current charge-up method, and the thermocouples and heating pads were attached to the surface of the proton battery stack to control the temperature. The instrument layout of the charge experiment is shown in Figure 2. The stereogram of the proton battery stack with the microsensor is shown in Figure 3.

When the hydrogen production rate in the water pipe in the sulfuric acid storage area was twice as high as the oxygen production rate in the A side upstream of the oxygen end, the active carbon inside the proton battery stack reached the upper bound of hydrogen absorption. At this point, the volume of gas collected via a drainage gas collection method could be recorded. The difference in the quantity of hydrogen was converted from the collected hydrogen and oxygen to obtain the mole number of gas, which was converted into weight. The weight percentage was converted using Equation (1):

(1) $$M\%=\frac{M}{M+M_{c}}\times 100\%$$

where M is the mass of hydrogen using the unit of grams, and M_c_ is the mass of the activated carbon using the unit of grams. In this study, the charge hydrogen storage capacity obtained from 0.8 g of microporous activated carbon at 30 °C, 40 °C, and 50 °C was 0.551, 0.549, and 0.434 wt%, respectively. The charging could be stopped when the current displayed by the power supply began to decrease from 0.1 A. The current-voltage values on both sides before and after charging at different temperatures were recorded, as shown in Table 2.

### 3.2. Internal Current Measurement during Proton Battery Stack Charging 

**Figure 4:**
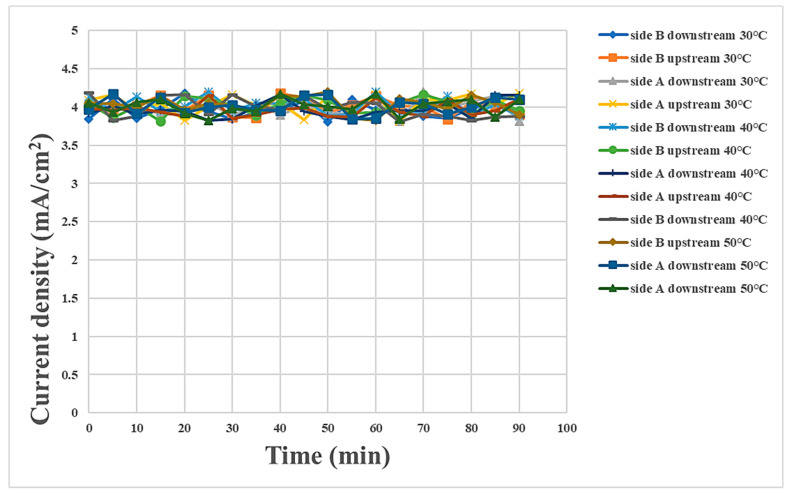
Internal current measurement during charging.

The proton battery stack was supplied with a stable constant current during charging in this experiment. As the measured internal current was in the same stable state, the internal electrochemical reaction was inferred to be stable, as shown in Figure 4.

### 3.3. Internal Flow Measurement of the Proton Battery Stack

**Figure 5:**
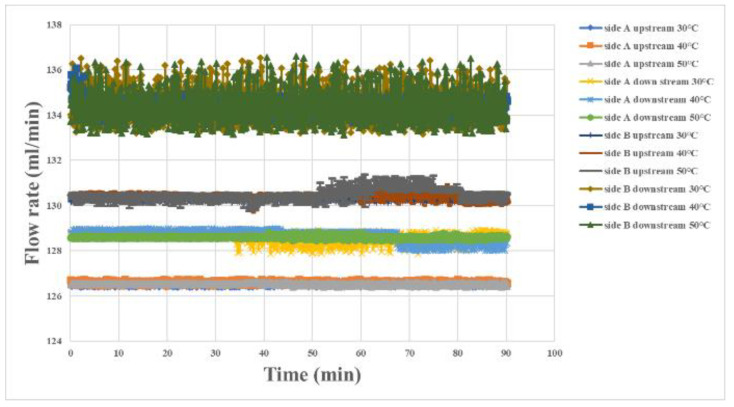
Internal flow measurement at the oxygen end of the proton battery stack.

The flowrate variation at the oxygen end of the proton battery stack is shown in Figure 5. As the carbon felt reduced during the flow, it could be observed that the flow decreased along the B side downstream, B side upstream, A side downstream, and the A side upstream in the direction of the flow. The severity of the flow rate variation matched the flow direction, where it was the most severe on the B side downstream at the water inlet, and the smoothest on the A side upstream at the water outlet.

### 3.4. Internal Humidity Measurement during Proton Battery Stack Charging 

**Figure 6:**
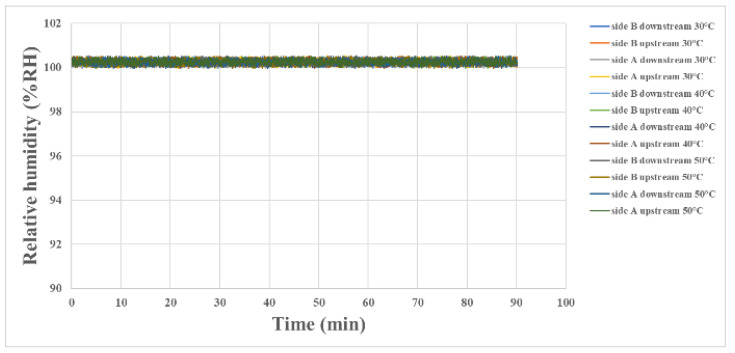
Internal humidity measurement at the oxygen end of the proton battery stack.

The internal humidity of the proton battery stack is shown in Figure 6. In the case of full water, the humidity upstream and downstream on each side was about 100%.

### 3.5. Internal Oxygen Sensing during Proton Battery Stack Charging

**Figure 7:**
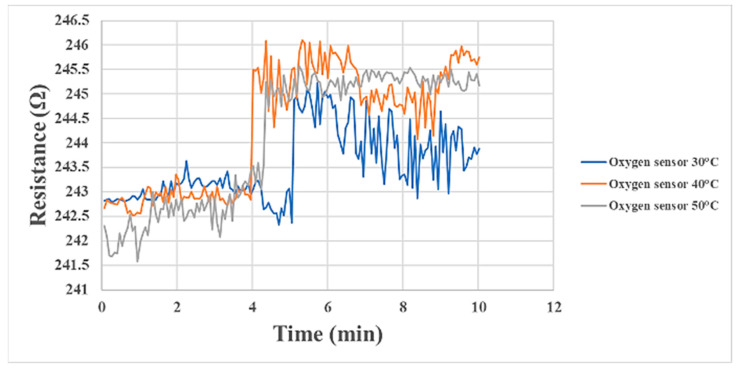
Internal oxygen sensing at the oxygen end of the proton battery stack.

Figure 7 shows oxygen sensing at 30 °C, 40 °C, and 50 °C during proton battery stack charging. As the half-reaction of the proton battery stack released oxygen, the oxygen-sensing time represents the reaction rate of the proton battery stack. It could be observed that the reaction at 40 °C was the fastest among the three temperatures, followed by 50 °C, and the reaction at 30 °C was the slowest. Therefore, it could be inferred that this proton battery stack membrane electrode assembly had the best performance at an operating temperature of 40–50 °C.

## 4. Internal Real-Time Microscopic Diagnosis of Proton Battery Stack during Discharging

**Figure 8:**
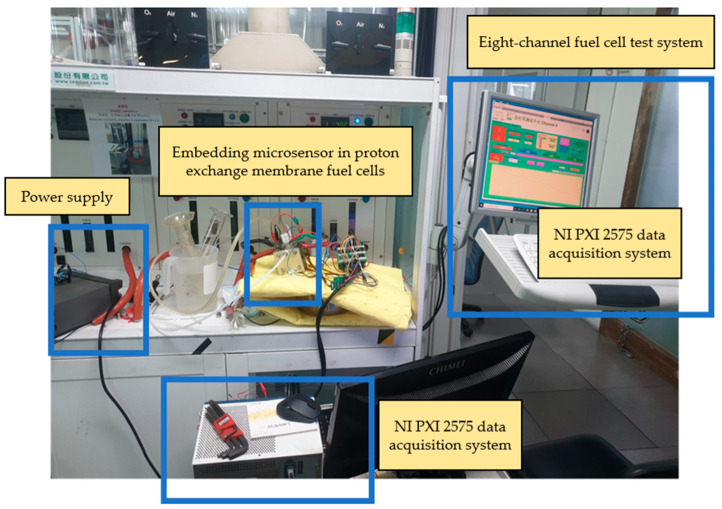
Proton battery stack discharge experiment.

The proton battery stack discharge was tested using an eight-channel fuel cell-testing machine. The experimental configuration for the discharge condition is shown in Figure 8. At 30 °C, 40 °C, and 50 °C and at a flow velocity of 500 mL/min, the air was admitted into the A-end upper opening at the oxygen end, respectively, for fuel cell reaction. The gas flew through the water pipe connected to the A-end lower opening to the B-end upper opening, and it was discharged out of the B-end lower opening connected to the flow pump in the charge experiment. Any large water globules inside the battery were removed by nitrogen before the experiment.

### 4.1. Proton Battery Stack Discharge Performance

**Figure 9:**
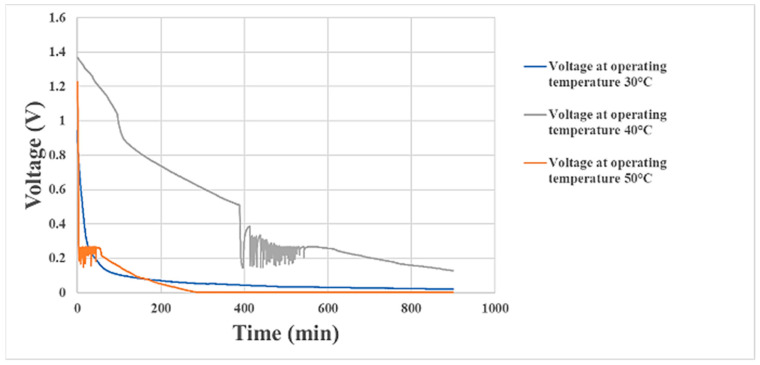
Voltage variation of the proton battery stack during discharging.

In the proton battery stack discharge experiment, a constant current of 0.1 A was applied to the stack, and the single cells on both sides were connected in parallel. The battery discharge voltage variation at three operating temperatures is shown in Figure 9. The hydrogen absorption/desorption capacities at the three temperatures were 0.212, 0.325, and 0.154 wt%. The drop in discharge voltage was abnormally rapid at 30 °C and 50 °C. The time consumption under normal discharge voltage at 40 °C was more than three times that of the other two temperatures. It was obvious that the suitable temperature for the membrane-electrode assembly during charging was also about 40 °C. The hydrogen absorption/desorption capacities at the three temperatures were compared and are shown in Table 3.

### 4.2. Internal Temperature Measurement during Proton Battery Stack Discharging

**Figure 10:**
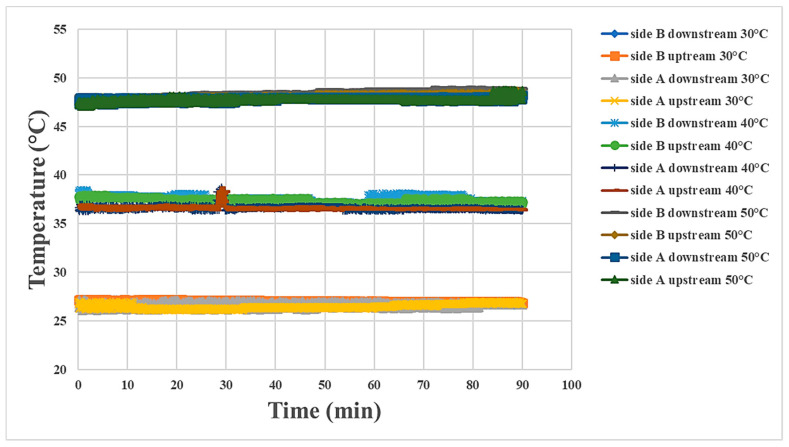
Temperature variation at the oxygen end of the proton battery stack.

**Figure 11:**
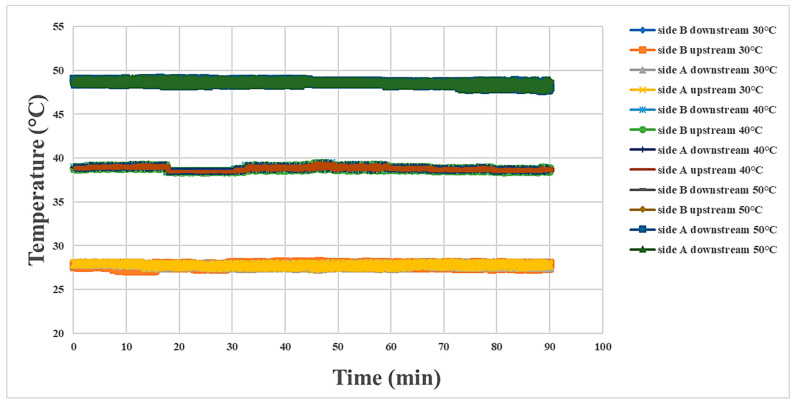
Temperature variation at the hydrogen end of the proton battery stack.

The internal temperatures of the proton battery stack measured at three temperatures are shown in Figure 10 and Figure 11. In comparison to the charging condition, the reaction was relatively weak during discharging, so the temperature variation was not obvious. The most severe temperature variation was at the oxygen end at 40 °C.

### 4.3. Internal Humidity Measurement during Proton Battery Stack Discharging

**Figure 12:**
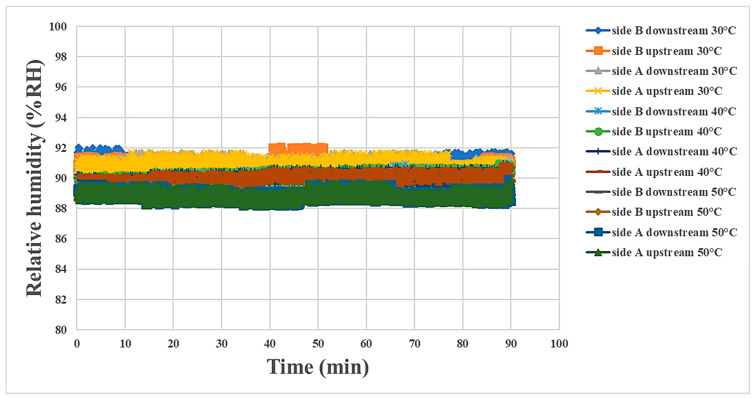
Humidity variation at the oxygen end of the proton battery stack.

In this study, the air was supplied at 90% humidity during discharging to assist the membrane reaction, and the humidity in various regions at the three temperatures was about 90%. The A side upstream contacted the air earliest, so the humidity was higher at this location than in the other regions. The B side downstream contacted the air last; hence, the humidity there was the lowest. The overall humidity decreased as the temperature increased, as shown in Figure 12. For the convenience of observation, the humidity was recorded every 15 min and is shown in Table 4.

### 4.4. Internal Flow Measurement during Proton Battery Stack Discharging

**Figure 13:**
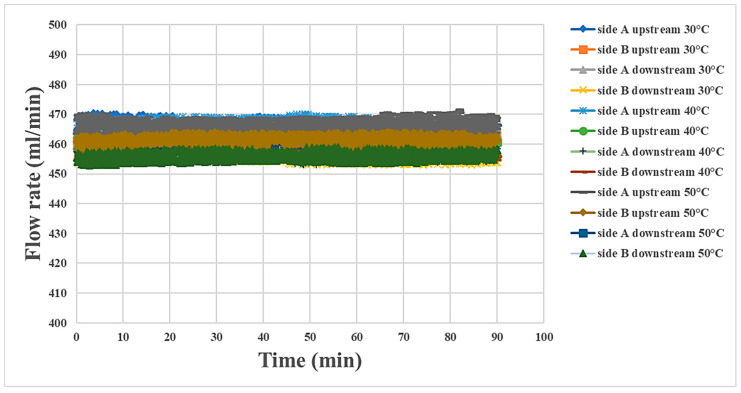
Flowrate variation at the oxygen end of the proton battery stack.

The proton battery stack was supplied with air at 500 mL/min at three temperatures for the discharge reactions. The flow measurement in various regions at the three temperatures is shown in Figure 13. The findings are similar to the trend for humidity, with the flow at the inlet (A side upstream) being the highest and that at the outlet (B side downstream) being the lowest. As the carbon felt allowed the gases to pass through, the decrease in the airflow was less severe than that of the water flow. For the convenience of observation, the flow rate was recorded every 15 min and is shown in Table 5.

## 5. Relationship between Internal Physical Quantities of Proton Battery Stack during Charging/Discharging and Temperature

**Figure 14:**
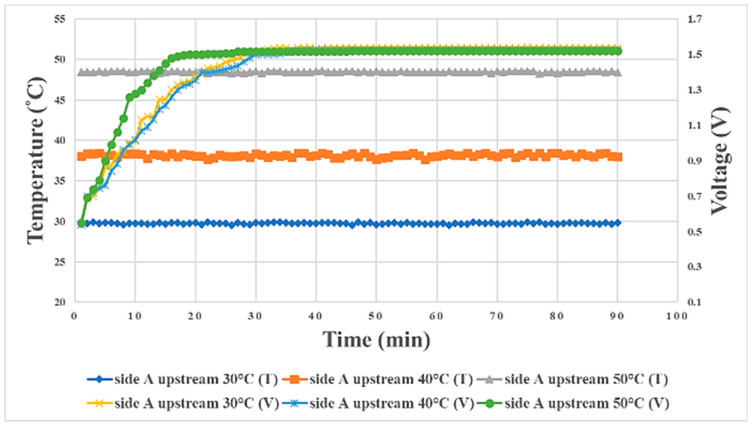
Graph of the relationship between voltage and temperature in the proton battery stack charge experiment, taking A side upstream as an example.

**Figure 15:**
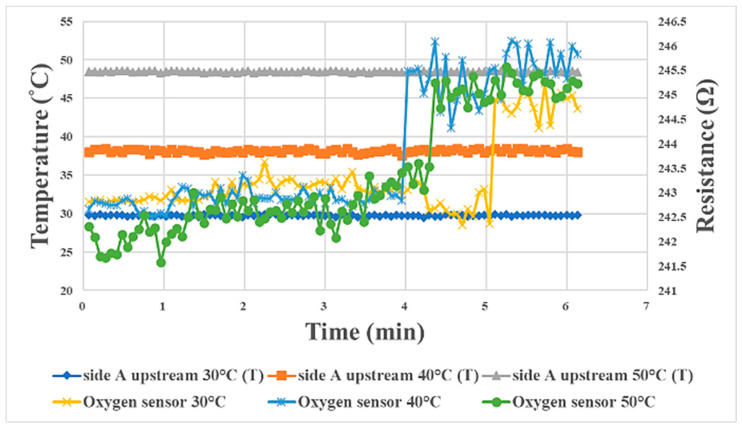
Graph of the relationship between oxygen and temperature in the proton battery stack charge experiment, taking A side upstream as an example.

**Figure 16:**
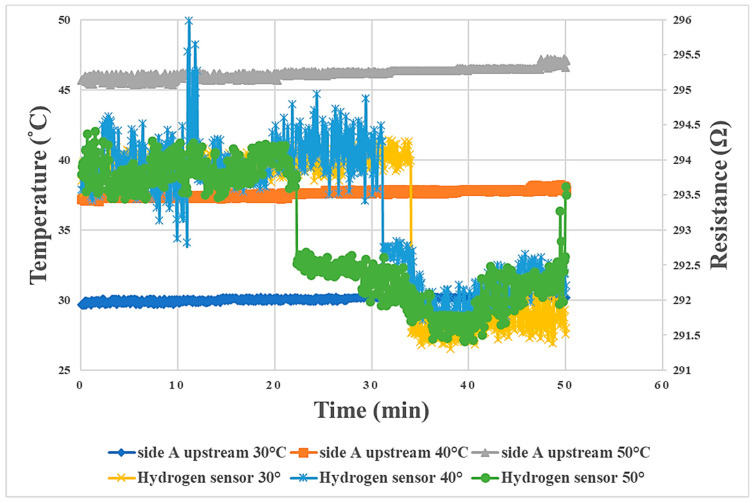
Graph of the relationship between hydrogen and temperature in the proton battery stack charge experiment, taking A side upstream as an example.

**Figure 17:**
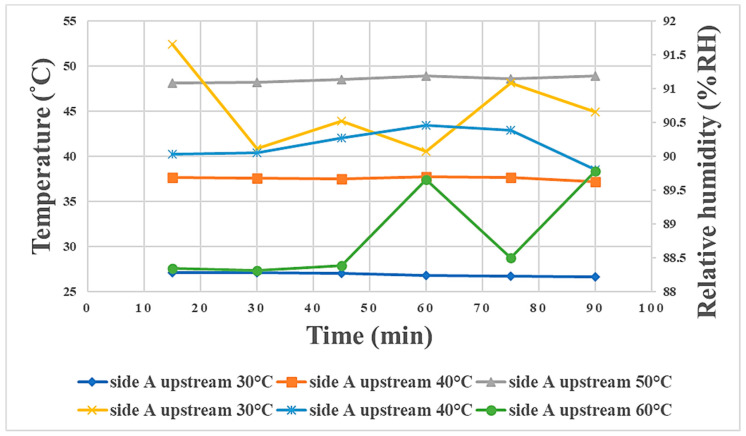
Graph of the relationship between humidity and temperature in the proton battery stack discharge experiment, taking A side upstream as an example.

In terms of the relationship between the internal physical quantities of the proton battery stack during charging/discharging and temperature, the A side upstream is taken as an example. The higher the temperature was during charging, the earlier the voltage was stabilized, and the final steady value was about 1.5 V, as shown in Figure 14. The best temperature found for this proton exchange membrane in this study was 40 °C, so the experimental temperature at which oxygen release was first detected was 40 °C. The reaction rate at 40 °C was the highest among the three temperatures, followed by 50 °C, with 30 °C being the lowest, as shown in Figure 15. The time for hydrogen release could be regarded as the hydrogen storage capacity of activated carbon. In this study, it was found that a higher temperature was less favorable for hydrogen storage using activated carbon. Therefore, the temperature at which the hydrogen was detected earliest was 50 °C, followed by 40 °C, and hydrogen was detected last at 30 °C, as shown in Figure 16. In the discharge experiment, when 90% RH air was supplied, the higher the temperature was, the lower the overall humidity was, as shown in Figure 17.

## 6. Conclusions

This study is an extension of the creation of a flexible six-in-one (hydrogen, voltage, current, temperature, humidity, and flow) microsensor [20]. In this study, a flexible seven-in-one (oxygen, hydrogen, voltage, current, temperature, humidity, and flow) microsensor was developed on a 50 µm polyimide (PI) film substrate by using MEMS technology for low thickness, good softness, a small area, corrosion resistance, and chemical resistance. The flexible seven-in-one microsensor was successfully embedded within a proton battery stack, and the actual data of the important physical quantities inside the proton battery stack during charging/discharging were extracted. In terms of the proton battery stack, the previously developed proton battery stack was subjected to detailed modifications to reduce the common problems of poor contact and leakage regarding the gas diffusion layer as per our prior experiments. During the charging/discharging of the proton battery stack, three temperatures were used for testing, and the most suitable temperature for the operation of this proton battery stack was found. The results show that higher temperatures were less favorable for hydrogen absorption using activated carbon. However, the charge/discharge performance of the membrane electrode assembly at 40 °C was better than that at 30 °C and 50 °C. Therefore, after careful consideration, the best temperature for the operation of this proton battery stack is 40 °C.

## Figures and Tables

**Table 1:** The developed microsensor and sensing area.

Sensor	Sensing Area
Micro voltage sensor	300 μm × 300 μm
Micro current sensor	300 μm × 300 μm
Micro temperature sensor	390 μm × 390 μm
Micro flow sensor	390 μm × 390 μm
Micro humidity sensor	390 μm × 390 μm
Micro hydrogen sensor	390 μm × 390 μm
Micro oxygen sensor	390 μm × 390 μm

**Table 2:** Initial and final voltages on both sides during the operation of the proton battery stack at three temperatures.

Temperature	30 °C	40 °C	50 °C
Initial voltage on side A	0.1 V	0.1 V	0.1 V
Final voltage on side A	1.52 V	1.56 V	1.53 V
Initial voltage on side B	0.1 V	0.1 V	0.1 V
Final voltage on side B	1.51 V	1.53 V	1.51 V

**Table 3:** Hydrogen absorption/desorption capacity of activated carbon in charge/discharge experiment.

Temperature	30 °C	40 °C	50 °C
Hydrogen uptake	0.551 wt%	0.549 wt%	0.434 wt%
Hydrogen discharge	0.212 wt%	0.325 wt%	0.154 wt%

**Table 4:** Humidity changes in the oxygen side of the proton cell stack (every 15 min).

	15 min	30 min	45 min	60 min	75 min	90 min
side B downstream 30 °C	91.17% RH	91.24% RH	90.64% RH	90.65% RH	91.04% RH	91.62% RH
side B upstream 30 °C	90.51% RH	90.50% RH	91.52% RH	91.26% RH	90.54% RH	90.95% RH
side A downstream 30 °C	90.21% RH	90.19% RH	91.11% RH	90.38% RH	90.62% RH	91.21% RH
side A upstream 30 °C	91.65% RH	90.11% RH	90.52% RH	90.07% RH	91.08% RH	90.66% RH
side B downstream 40 °C	89.27% RH	89.99% RH	89.75% RH	89.13% RH	90.60% RH	90.42% RH
side B upstream 40 °C	89.57% RH	89.95% RH	90.42% RH	89.65% RH	90.32% RH	90.35% RH
side A downstream 40 °C	90.26% RH	90.37% RH	90.39% RH	90.48% RH	90.07% RH	89.93% RH
side A upstream 40 °C	90.03% RH	90.05% RH	90.27% RH	90.46% RH	90.38% RH	89.80% RH
side B downstream 50 °C	89.46% RH	88.98% RH	88.30% RH	89.18% RH	88.69% RH	88.91% RH
side B upstream 50 °C	89.55% RH	89.38% RH	88.63% RH	88.72% RH	89.04% RH	89.39% RH
side A downstream 50 °C	88.38% RH	89.12% RH	88.52% RH	88.68% RH	88.67% RH	88.47% RH
side A upstream 50 °C	88.35% RH	88.31% RH	88.39% RH	89.66% RH	88.50% RH	89.78% RH

**Table 5:** Changes in the flow rate at the oxygen end of the proton battery stack (every 15 min).

	15 min	30 min	45 min	60 min	75 min	90 min
side A upstream 30 °C	466.19 mL/min	465.21 mL/min	464.87 mL/min	465.73 mL/min	464.66 mL/min	463.74 mL/min
side B upstream 30 °C	459.78 mL/min	461.70 mL/min	462.68 mL/min	463.35 mL/min	462.69 mL/min	460.75 mL/min
side A downstream 30 °C	457.60 mL/min	458.95 mL/min	458.95 mL/min	458.48 mL/min	457.26 mL/min	457.20 mL/min
side B downstream 30 °C	457.88 mL/min	456.45 mL/min	457.55 mL/min	456.81 mL/min	453.97 mL/min	453.74 mL/min
side A upstream 40 °C	465.64 mL/min	468.13 mL/min	466.30 mL/min	464.45 mL/min	466.17 mL/min	464.86 mL/min
side B upstream 40 °C	461.82 mL/min	461.32 mL/min	461.90 mL/min	463.73 mL/min	462.52 mL/min	460.23 mL/min
side A downstream 40 °C	456.47 mL/min	456.44 mL/min	456.25 mL/min	456.04 mL/min	454.42 mL/min	458.48 mL/min
side B downstream 40 °C	457.44 mL/min	454.27 mL/min	455.26 mL/min	456.29 mL/min	455.69 mL/min	455.82 mL/min
side A upstream 50 °C	465.14 mL/min	468.29 mL/min	468.90 mL/min	469.18 mL/min	469.21 mL/min	466.38 mL/min
side B upstream 50 °C	462.33 mL/min	462.32 mL/min	462.28 mL/min	459.89 mL/min	463.33 mL/min	461.20 mL/min
side A downstream 50 °C	457.15 mL/min	454.79 mL/min	456.30 mL/min	453.68 mL/min	457.58 mL/min	457.86 mL/min
side B downstream 50 °C	455.67 mL/min	458.35 mL/min	459.11 mL/min	453.67 mL/min	456.70 mL/min	455.88 mL/min

## Data Availability

Not applicable.
